# Efficacy and tolerability of Janus kinase inhibitors in myelofibrosis: a systematic review and network meta-analysis

**DOI:** 10.1038/s41408-021-00526-z

**Published:** 2021-07-27

**Authors:** Léa Sureau, Corentin Orvain, Jean-Christophe Ianotto, Valérie Ugo, Jean-Jacques Kiladjian, Damien Luque Paz, Jérémie Riou

**Affiliations:** 1 Univ Angers, Inserm, CRCINA, Angers, France; 2 CHU Angers, Laboratoire d’hématologie, Angers, France; 3 Fédération Hospitalo-Universitaire ‘Grand Ouest Against Leukemia’ (FHU GOAL), Angers, France; 4 CHU Angers, Service des maladies du sang, Angers, France; 5 CHRU Brest, Hématologie Clinique, Institut de Cancéro-Hématologie, Brest, France; 6 Université de Paris, APHP, Hôpital Saint-Louis, Centre d’Investigations Cliniques, INSERM CIC 1427, Paris, France; 7 Univ Angers, INSERM, UMR 1066, CNRS 6021, MINT, Angers, France; 8 CHU Angers, DRCI, Département de biostatistiques et méthodologie, Angers, France

**Keywords:** Molecularly targeted therapy, Targeted therapies

## Abstract

Myelofibrosis is a myeloproliferative neoplasm associated with constitutional symptoms, increasing splenomegaly, and worsening cytopenias. Janus kinase (JAK) inhibitors have been used for the treatment of myelofibrosis for several years, but there is a lack of comparative information between those treatments. A systematic review and network meta-analysis was performed on randomized controlled trials in patients with myelofibrosis receiving JAK inhibitor or placebo or control. Primary outcomes were efficacy on spleen volume reduction and total symptom score reduction. Additional analyses were conducted on anemia and thrombopenia events. Seven studies were included in the network meta-analysis including 1953 patients randomly assigned to four JAK inhibitors—ruxolitinib, fedratinib, pacritinib, momelotinib—or control. In first-line therapy, momelotinib and fedratinib were associated with comparable efficacy to ruxolitinib, and with less toxicity on erythrocytes and platelets, respectively. Pacritinib was less effective on splenomegaly than ruxolitinib as a first-line treatment but seemed effective in second line, after ruxolitinib exposure. Fedratinib and ruxolitinib that are FDA approved in myelofibrosis have both confirmed being valuable option to treat splenomegaly and constitutional symptoms, and their slightly different tolerance-profiles can guide therapeutic choice for first-line treatment, according to patient profile. Momelotinib could be another option especially due to its positive effect on anemia.

## Introduction

Myeloproliferative neoplasms (MPN) are acquired clonal disorders characterized by a proliferation and an accumulation of mature blood cells that include polycythemia vera (PV), essential thrombocythemia (ET), and primary myelofibrosis (PMF). Myelofibrosis (MF) is characterized by a proinflammatory signature and a dysregulation of the bone marrow stroma with the development of a reticulin fibrosis. The annual incidence rate ranges from 0.22 to 0.99 per 100,000 for PMF with a median age at diagnosis of 65 years [1]. Primary myelofibrosis differs from post-PV or post-ET secondary myelofibrosis (SMF) with a rate of evolution around 10% after 10 years of follow-up [2]. The course of myelofibrosis is associated with progressive constitutional symptoms (e.g.: fatigue, night sweats, and fever), increasing splenomegaly, and worsening cytopenias. Among myeloproliferative neoplasms, myelofibrosis is associated with the worst prognosis with a median overall survival between 13 months and 11 years according to prognostic features and management is extremely variable, from watch-and-wait strategy to bone marrow transplantation [3]. Main causes of mortality include leukemic transformation, bleeding or infections related to cytopenias and cardiovascular events [3].

In almost all cases, MPN harbor somatic mutations in driver genes *JAK2*, *CALR*, or *MPL* that lead to a constitutive activation of the JAK-STAT pathway. Thus, the inhibition of the JAK2 tyrosine kinase appeared to be a promising target for drug development. Ruxolitinib was the first JAK1/JAK2 inhibitor approved by the US Food and Drug Administration (FDA) in intermediate-2 and high-risk myelofibrosis. Ruxolitinib is effective in decreasing splenomegaly and improving patients’ symptoms [4, 5]. Moreover, recent long-term follow-up of COMFORT I/II clinical trials suggest a benefit on overall survival for ruxolitinib-treated patients [6]. Recently, a second JAK2-only inhibitor, fedratinib, has been FDA approved for first-line and second-line therapy after ruxolitinib failure and other JAK1-JAK2 (momelotinib) or JAK2-only (pacritinib) inhibitors are in phase 3 development [7]. All JAK inhibitors have shown a significant effect on splenomegaly reduction compared to either placebo, best available treatment or ruxolitinib in different clinical trials. Interestingly, tolerance and adverse events seem to be different according to JAK inhibitors. However, there is a lack of data regarding direct comparison of anti-JAKs to each other. The aim of our study, using a systematic review and a network meta-analysis, was to evaluate and compare JAK inhibitors for their efficacy and tolerability.

## Methods

### Study selection

Eligible studies for this meta-analysis were placebo-controlled and head-to-head, randomized controlled trials (RCTs) to prevent selection bias according to Cochrane Handbook for Systematic Reviews and Interventions [8].

A search of studies of interest was carried out on PubMed, Cochrane Central Register of Controlled Trials (CENTRAL), ClinicalTrials.gov and abstracts and presentations from major hematological meeting without language restrictions from database inception until April 12, 2021. The search strategy combined the keywords “myelofibrosis” and “JAK inhibitor” (or synonyms), and collected studies were then manually checked for consistency with inclusion criteria (see supplementary materials for details, Figs. S1 and S2 and Tables S1 and S2). Two investigators (LS and DLP) independently reviewed the titles, abstracts, and study designs to establish whether they met the inclusion criteria. Internal validity of included studies was evaluated, as recommended by Cochrane Handbook [8], with a plot which summarizes the potential bias presented in supplementary materials (Fig. S3).

RCTs including patients with primary or secondary myelofibrosis evaluating safety and efficacy of any JAK inhibitor within a time frame of at least 24 weeks were included (see flow chart in supplementary materials). Studies related to investigational medicinal products not belonging to the therapeutic class of JANUS kinases inhibitors were excluded, as well as trials in which JAK inhibitors were used in combination with another treatment, trials where the main condition of the patients was not exclusively primary and secondary myelofibrosis or trials evaluating JAK inhibitors before allogenic stem cell transplantation.

### Endpoints, definitions, and study populations

The primary endpoint was a spleen volume reduction (SVR) upper than 35 percent after 24 weeks of treatment. Secondary endpoints included the total symptom score reduction (TSSR) evaluated using the Myelofibrosis Symptom Assessment Form (MF SAF) 2.0 [9], and main adverse events due to hematologic toxicity, i.e., grade 3 or 4 anemia and grade 3 or 4 thrombocytopenia over the 24 weeks of treatment—defined-, respectively, for grade 3 as an hemoglobin rate < 8 g/dL and a platelets count < 50.10^9^/L, as per the Common Terminology Criteria for Adverse Events v4.0 (CTCAE) [10].

The definitions of each endpoint as applied in each trial were incorporated. Main analyses were performed in the intention-to-treat populations, except for TSSR analyses which were on evaluable populations. This was consistent with the methods of all included trials, where patients included in TSSR analysis were those with TSSR values available at baseline and week 24. Studies registration numbers and names of registries are detailed in the supplementary materials.

The present review was performed according to PRISMA statements (Supplementary Table S3) [11, 12].

### Statistical analysis

Both frequentist and a Bayesian framework with noninformative prior approaches were performed for all endpoints’ analysis [13]. Odds Ratio (OR) and 95% confidence intervals (CIs) were used as the summary statistic. Estimates of Odds for each treatment and endpoints were extracted from the main publications of RCTs, obtained from principal investigators, or calculated as previously described [14]. The pooled OR was calculated with both fixed effect and random effect models.

The extent of small study effects and publication bias were assessed by visual inspection of funnel plots [15]. The heterogeneity across trials was evaluated with the I² statistic; less than 25% represented low heterogeneity, 25–50% represented mild heterogeneity, 50–75% represented moderate heterogeneity, and higher than 75% represented severe heterogeneity [16]. Sensitivity analyses were performed by assessing the effect of removing individual studies when heterogeneity across trials was observed.

Consistency of inferential estimates were also appraised with a Bayesian framework, computing OR and 95% credible intervals (CrI) with a hierarchical model by means of Markov Chain Monte Carlo (MCMC) methods with Gibbs sampling from 10.000 iterations obtained after a 1000-iterations training phase. The convergence was estimated according to density plot, trace plot and the Brooks-Gelman-Rubin method [17, 18]. MCMC simulations were performed by means of JAGS software in R by use of gemtc and rjags package [19].

The Surface Under the Cumulative RAnking (SUCRA) score and forest plots, were performed to evaluate and summarize the main results [20]. All statistical analyses were performed using R software version 3.6.2 [21].

## Results

We identified a total of 162 articles referring to clinical trials possibly eligible for this study. In the end, seven studies were included in the meta-analysis, all of them evaluating one of the following JAK inhibitors: ruxolitinib, momelotinib, fedratinib and pacritinib (Supplementary Figs. S1 and S2). The characteristics of these seven trials are summarized in Table 1.

**Table 1:** Trials of anti-JAK2 efficacy and safety meeting inclusion criteria.

	Number of patients enrolled in each arm	Primary endpoint	Previous ruxolitinib exposure	Platelets count at baseline	Minimal treatment period	Results
COMFORT-1 [37]	Ruxolitinib (*n* = 155)	SVR	No	≥100.10^9^/L	24 weeks	Ruxolitinib was superior to placebo for spleen response (*p* < 0.001).
	Placebo (*n* = 154)					
COMFORT-2 [5]	Ruxolitinib (*n* = 146)	SVR	No	≥50.10^9^/L	48 weeks, data available at 24 weeks	Ruxolitinib was superior to BAT for spleen response (*p* < 0.001).
	BAT (*n* = 73)					
JAKARTA-1 [38]	Fedratinib 400 mg daily (*n* = 96)	SVR	No	≥50.10^9^/L	24 weeks	Fedratinib (all arms) was superior to placebo for spleen response (*p* < 0.001).
	Fedratinib 500 mg daily (*n* = 97)					
	Placebo (*n* = 96)					
PERSIST-1 [39]	Pacritinib (*n* = 220)	SVR	No	Not specified	24 weeks	Pacritinib was superior to BAT including watchful waiting for spleen response (*p* = 0.0003).
	BAT excluding anti JAK (*n* = 107)					
PERSIST-2 [40]	Pacritinib 400 mg once daily (*n* = 104 (75^a^))	SVR, total symptom score reduction	Previous ruxolitinib exposure or not	Must be <100.10^9^/L	24 weeks	Pacritinib (all arms) was superior to BAT including ruxolitinib for spleen response (*p* = 0.001) and for symptom response (*p* = 0.08).
	Pacritinib 200 mg twice daily (*n* = 107 (74^a^))					
	BAT (*n* = 100 (72^a^))					
SIMPLIFY-1 [28]	Momelotinib (*n* = 215)	SVR	No	≥50.10^9^/L	24 weeks	Momelotinib was noninferior to ruxolitinib for spleen response (*p* = 0.011) but not for symptom response (*p* = 0.98). Momelotinib treatment was associated with a reduced transfusion requirement (*p* < 0.019).
	Ruxolitinib (*n* = 217)					
SIMPLIFY-2 [27]	Momelotinib (*n* = 104)	SVR	Yes, after ruxolitinib exposure	Not specified	24 weeks	Momelotinib was not superior to BAT for spleen response (*p* = 0.90) but was superior in symptom response (*p* = 0.0006).
	BAT (*n* = 52)					

*SVR* spleen volume reduction, *BAT* best available therapy.

^a^Only patients who completed at least 22 weeks of follow-up after randomization and before clinical hold were taken into account.

A total of 1953 patients were included in the analysis. Participants were adults with PMF (58.3%), post-PV (25.4%), or post-ET myelofibrosis (16.2%) or missing (0.1%). For each endpoint, trials underwent a new selection process to be part of the analysis, depending on their specificities, especially the possibility for the participants to have been exposed to ruxolitinib prior to the trial.

### Efficacy

**Fig. 1:**
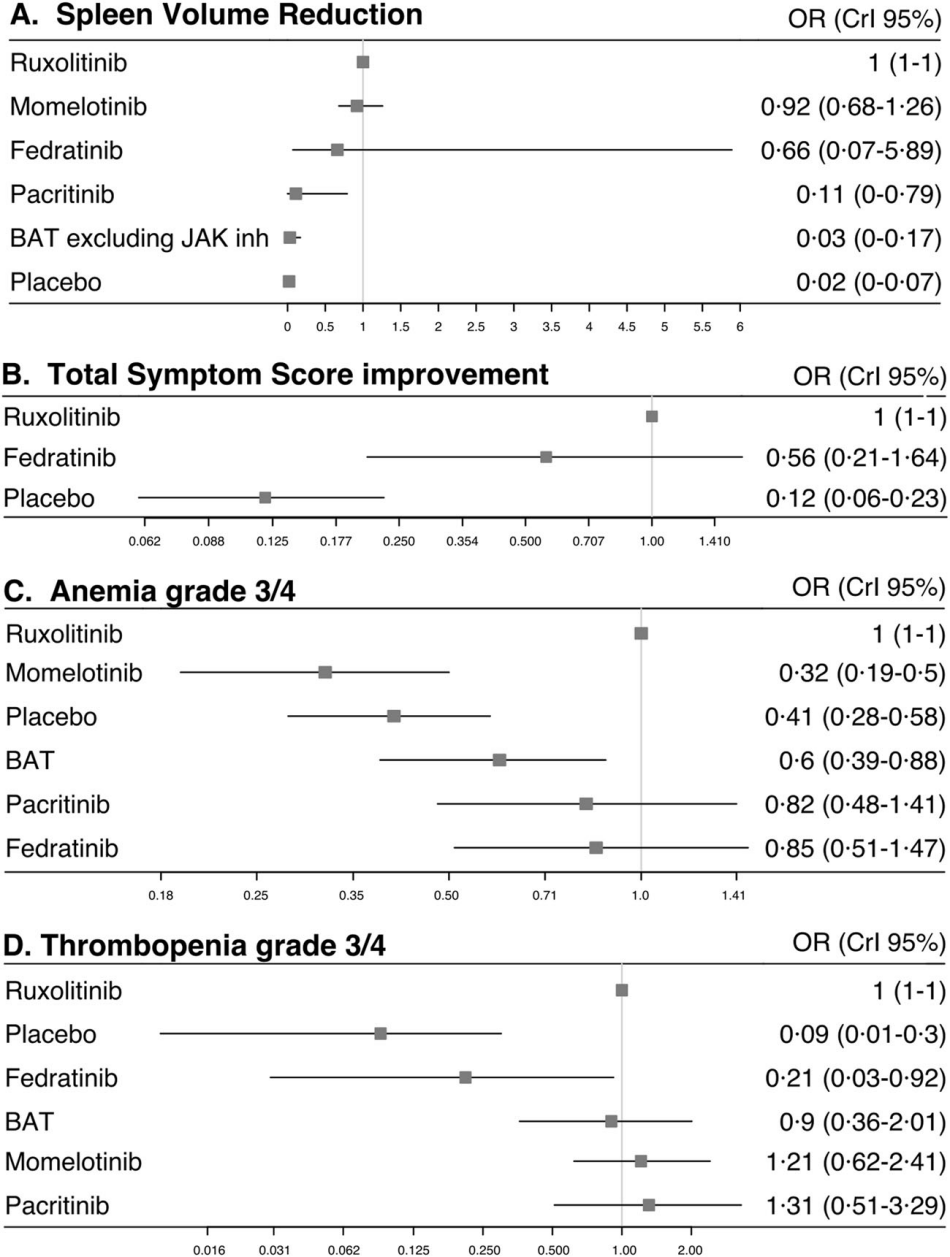
Forest plots of odds ratio (OR) for efficacity and toxicity endpoints of JAKi. Estimates of risk in the intention-to-treat population for **A** spleen volume reduction, in the per protocol population for **B** total symptom score reduction, in the intention-to-treat population for **C** grade 3/4 anemia events, and for **D** grade 3/4 thrombocytopenia events.

Regarding the primary endpoint of a SVR of at least 35 percent at 24 weeks, moderate heterogeneity appeared across trials (I^2^ = 67.9% [0.0%; 90.7%]), which was due to the mix of data referring to first-line and second-line treatments as shown by the funnel plot (Supplementary Fig. S4). Bayesian network meta-analysis was therefore performed with first-line data exclusively, excluding SIMPLIFY-2 and PERSIST-2 trials that included second-line participants (Table 1). Thus, five studies (71%) with 1576 patients (81%) were included in this analysis showing that fedratinib, momelotinib, and ruxolitinib were associated with a significant improvement in SVR compared to placebo. Ruxolitinib and momelotinib proved to be associated with a significant improvement in primary endpoint achievement in comparison to pacritinib, whereas fedratinib failed to prove the same. However, no statistically significant difference was demonstrated between fedratinib, momelotinib, and ruxolitinib on the SVR criterion (Fig. 1A).

A sensitivity test was performed with all trials data, combining first-line and second-line patients (Supplementary Fig. S5). For this analysis, trials reports allowed for the segregation of patients treated with ruxolitinib as BAT in PERSIST-2 and SIMPLIFY-2 studies. These two trials were thus considered as three-arms studies: pacritinib or momelotinib vs BAT (excluding ruxolitinib) vs ruxolitinib. This analysis showed a greater efficacy for pacritinib in comparison to ruxolitinib, although not reaching statistical significance. Results obtained for fedratinib, momelotinib, and BAT were similar with those from the main analysis.

The secondary efficacy endpoint of TSS reduction by at least 50% at 24 weeks was conducted with the only two studies JAKARTA-1 and COMFORT-1 (with 567 patients) that used the MF SAF v2.0 score to ensure data consistency. Bayesian analysis showed that both ruxolitinib and fedratinib were associated with a significant improvement in symptom, in comparison to placebo. No difference was found between ruxolitinib and fedratinib on the TSSR criterion (Fig. 1B).

For both efficacy endpoints, the results were confirmed using the frequentist method, i.e., the second method for network meta-analysis.

### Safety

The analysis on grade 3/4 anemia events during JAK inhibitor therapy was conducted with data from all seven studies (1953 patients) mixing first-line and second-line treatments, with a moderate heterogeneity across trials (I^2^ = 54% [0.0%; 86.8%]). Bayesian network meta-analysis showed significantly less grade 3/4 anemia with momelotinib than with ruxolitinib, fedratinib, or pacritinib. Analysis did not show any statistically significant difference between ruxolitinib, fedratinib, and pacritinib (Fig. 1-C). This was confirmed by two sensitivity analyses conducted without trials for which overall participants hemoglobin rate was significatively different: one without SIMPLIFY-2 in which more patients had anemia at baseline, and the second without PERSIST-2 in which fewer patients had anemia at baseline. An additional sensitivity analysis performed with trials in first-line setting only showed the same results. Of note, this sensitivity analysis does not consider trials that did not exclude JAK inhibitors from their BAT (Supplementary Fig. S6, Supplementary Table S4).

The occurrence of grade 3/4 thrombocytopenia events over the 24 weeks with JAK inhibitor therapy, was also analyzed using all seven trials with a mild heterogeneity across trials (I^2^ = 32.8% [0.0%; 93.0%]). Bayesian network meta-analysis demonstrated fewer occurrence of thrombocytopenia with fedratinib compared to ruxolitinib, momelotinib, and pacritinib. Analysis did not highlight any statistically significant difference between ruxolitinib, momelotinib, and pacritinib (Fig. 1D). A sensitivity analysis was performed without studies that included participants with thrombocytopenia <50.10^9^/L at baseline (i.e., SIMPLIFY-2, and the PERSIST-1 and PERSIST-2 trials with pacritinib, Table 1) and hence using first-line data only. This analysis confirmed the lower risk of thrombocytopenia with fedratinib compared to ruxolitinib and momelotinib (Supplementaryl Fig. S7, Supplementary Table S5).

Results of analyses performed using the frequentist method were consistent with those obtained with the bayesian method.

## Discussion

The systematic review of randomized controlled trials led to the inclusion of seven trials and 1953 patients in this meta-analysis, allowing to obtain new data about the relative efficacy and safety of the different JAK2 inhibitors by indirect comparison.

In the absence of a direct comparison between the two JAK inhibitors currently approved for the treatment of myelofibrosis; our results confirm that fedratinib is a solid alternative to ruxolitinib. Indeed, this analysis showed that fedratinib was not inferior to ruxolitinib for reducing splenomegaly and improving symptoms. Among investigational agents, momelotinib as first-line or second-line therapy after ruxolitinib appears to be a valuable option to reduce splenomegaly, although it was not possible to obtain results on its relative efficacy on constitutional symptoms. Concerning pacritinib, our results showed better efficacy to reduce spleen volume in patients with prior ruxolitinib exposure than in patients naïve to JAK inhibitors. Other JAK inhibitors may also be useful after ruxolitinib failure. For example, a recent update of the fedratinib JAKARTA-2 study (NCT01523171) focusing on patients with a stringent definition of prior ruxolitinib failure with an intention-to-treat analysis showed 30% of SVR with a median duration of response not reached [22].

The most frequent reasons leading to ruxolitinib discontinuation are loss of response and hematologic toxicity [23, 24]. Additional analyses performed on safety data may therefore tip the balance in favor of one specific JAK inhibitor.

Both primary analysis and sensitivity tests showed that patients treated with momelotinib exhibited less grade 3/4 anemia than those treated with other JAK inhibitors, and also than patients who received placebo. The effect of momelotinib on anemia could be explained by its capacity to decrease hepcidin production [25, 26]. This result is reinforced by the fact that in both studies investigating momelotinib, more patients had anemia at baseline compared to the other studies. In the SIMPLIFY-2 study, including patients treated with ruxolitinib who either required RBC transfusion, had grade 3 anemia or thrombocytopenia, or grade 3/4 bleeding, momelotinib was not superior to BAT but there was a higher proportion of transfusion-independent patients in the momelotinib arm (43 *vs* 21%) [27]. Of note, patients were not stratified at randomization on the prior spleen response with ruxolitinib and there was no ruxolitinib-washout period before study entry. Thus, our meta-analysis supports previous observations making momelotinib a valuable option for reducing splenomegaly and improving anemia [28, 29]. More insight on the effects of momelotinib in patients with myelofibrosis and anemia will be gained with the ongoing MOMENTUM trial (NCT04173494) [30]. It will compare the effectiveness of momelotinib to danazol in treating and reducing disease-related symptoms, requirement for blood transfusions, and splenomegaly in anemic patients with MF. Another promising option for patients with anemia could be a dosing strategy as recently reported for ruxolitinib in the phase 2 REALISE study (NCT02966353) [31].

The results of this meta-analysis regarding the occurrence of grade 3/4 thrombocytopenia events showed that fedratinib was less toxic on platelets than ruxolitinib. Considering that our analysis failed to show a statistically significant difference on anemia events between fedratinib and ruxolitinib, and that their efficacy on splenomegaly and disease-related symptoms were not significantly different, our results suggest that fedratinib could be a valuable first-line therapy in ruxolitinib-naïve patients. The efficacy on both splenomegaly and constitutional symptoms in the second-line setting, after ruxolitinib was demonstrated in the JAKARTA-2 study, which was not included in this systematic review because of its single-arm design [22, 32]. Surprisingly, the analysis of thrombocytopenia has failed to show a statistically significant difference between pacritinib and ruxolitinib, even if PERSIST trials included patients with thrombocytopenia. Furthermore, the phase 3b JUMP study has confirmed the efficacy and safety of ruxolitinib in patients with low platelets count (NCT01493414), although treatment with ruxolitinib should be interrupted in patients with platelet counts <50.10^9^/L [33]. The good tolerance of pacritinib in this population still needs to be confirmed in a randomized trial. The PACIFICA study (NCT03165734), which is recruiting patients with MF and severe thrombocytopenia (platelet counts < 50.10^9^/L at baseline), will evaluate pacritinib as first- or second-line treatment after JAK inhibitor versus physician’s choice, that may include low-dose ruxolitinib. The phase 2 PAC203 study has demonstrated that the dose of 200 mg twice daily of pacritinib provided better response than low dosages with no excess of grade 3 events [34].

Alongside the hematologic toxicity, other toxicities should be taken into account to guide individual JAK inhibitors therapies. We compiled all adverse events reported in the clinical trials in Supplementary Table S6 showing that only few grade 3/4 nonhematological events were reported. The nonhematological toxicity profile seems to be different between the four JAK inhibitors with more gastro-intestinal events for fedratinib and pacritinib. Finally, some adverse events are more specific to some drugs with low-grade peripheral neuropathy reported for momelotinib or rare cardiac events for pacritinib. Of note, few cases of Wernicke encephalopathies (WE) occurred during fedratinib clinical trials that led to investigational suspension (a total of seven cases were suspected in several trials involving fedratinib). It has since been proved that an appropriate thiamine level could prevent WE [22]. Also, thiamine deficiency is rare in MPN patients regardless of therapy received but should be carefully investigated and supplemented before starting fedratinib therapy [35].

The main bias in this systematic review and meta-analysis was the low number of trials included mainly due to the relatively small number of comparative studies conducted in myelofibrosis. However, all outcomes included in these analyses were objectively assessed in the original trials: primary outcome of SVR at week 24 was assessed by a blinded central reader for all of the included studies, grade 3/4 anemia and thrombocytopenia events were defined as per the CTCAE v4.0 and TSS was evaluated with standardized questionnaires. For this latter endpoint, however, the existence of different instruments was a constraint by limiting the eligible studies for the analysis, and a gold standard should be set for future trials. Baseline characteristics of patients regarding disease-related risk levels (estimated with the IPSS or DIPSS [3, 36]) and ECOG performance status were not fully harmonized between included studies. JAKARTA-1, the only trial evaluating fedratinib in this meta-analysis, and COMFORT-1 and 2 studies excluded patients with intermediate-1 risk myelofibrosis. Also, only the two COMFORT trials allowed the inclusion of patients with an ECOG performance status superior to 2. The multiplication of prognostic scores in myelofibrosis, whether clinical or including cytogenetic/molecular data, and the number of potential stratification categories can be a limit in the future for the comparison between trials and interpretation of their results. In clinical trials design, one should be careful to maintain harmonization in the use of these parameters.

In conclusion, this study confirms some empirical observations regarding the relative efficacy and tolerance of the different JAK inhibitors available to treat patients with myelofibrosis. Altogether, our results support the place of ruxolitinib as the reference JAK inhibitor, closely followed by fedratinib, for reducing splenomegaly and improving disease-related symptoms. This study suggests that the choice of a JAK inhibitor could depend on the line of treatment and to the risk of onset of severe anemia and/or thrombocytopenia. In this regard, momelotinib could be confirmed as a valuable option in case of anemia and fedratinib in case of thrombocytopenia. Pacritinib should be confirmed as a valuable option in a second-line setting after prior JAK inhibitor exposure. In addition, future trials are necessary to assess the influence of additional parameters on the choice of a JAK inhibitor, like the mutational landscape and the role of nondriver mutations.

## Supplementary information

Supplementary data
