# Supplementary material for: Efficacy and tolerability of Janus kinase inhibitors in myelofibrosis: a systematic review and network meta-analysis
Source: Blood Cancer J. 2021 Jul 27;11(7):135. doi: 10.1038/s41408-021-00526-z (PMC8316412; doi:10.1038/s41408-021-00526-z)
Supplement: Supplementary file 1 — Supplementary data [file 41408_2021_526_MOESM1_ESM.docx]

Efficacy and tolerability of Janus kinase inhibitors in myelofibrosis: a systematic review and network meta-analysis

Supplemental materials

[Study selection 2](#_Toc76644704)

[Summary of the risk of bias in the included studies 3](#_Toc76644705)

[Preliminary and sensitivity tests for Spleen Volume Reduction (SVR) 4](#_Toc76644706)

[Sensitivity tests and additional analysis for grade 3/4 anemia events 5](#_Toc76644707)

[Sensitivity test for grade 3/4 thrombopenia events 7](#_Toc76644708)

[Study screening and inclusion in the systematic review and meta-analysis 8](#_Toc76644709)

[PRISMA checklist for systematic review or meta-analysis 9](#_Toc76644710)

[Baseline repartition depending on hemoglobin rate and platelet counts 12](#_Toc76644711)

[Adverse events of JAK inhibitors 14](#_Toc76644712)

# Study selection

Records identified through searching (n=162)

Studies screened (n=113)

Studies assessed for eligibility and quality (n=7)

Studies included in systematic review and meta-analysis (n=7)

Duplicate studies removed based on record title, PubMed UID or related NCT (n=49)

Studies excluded as they did not meet the inclusion criteria (n=106)

Retrospective or observational study (n=7)

Non-randomized study (n=24)

Myelofibrosis is not an inclusion criterion (n=21)

IMP is not an anti-JAK (n=34)

Outcome is irrelevant (n=20)

**Figure S1**: Flow of the study selection for the systematic review

IMP: Investigational Medicinal Product; JAK: Janus Kinase; NCT: ClinicalTrials.gov identifier; UID: Unique Identifier


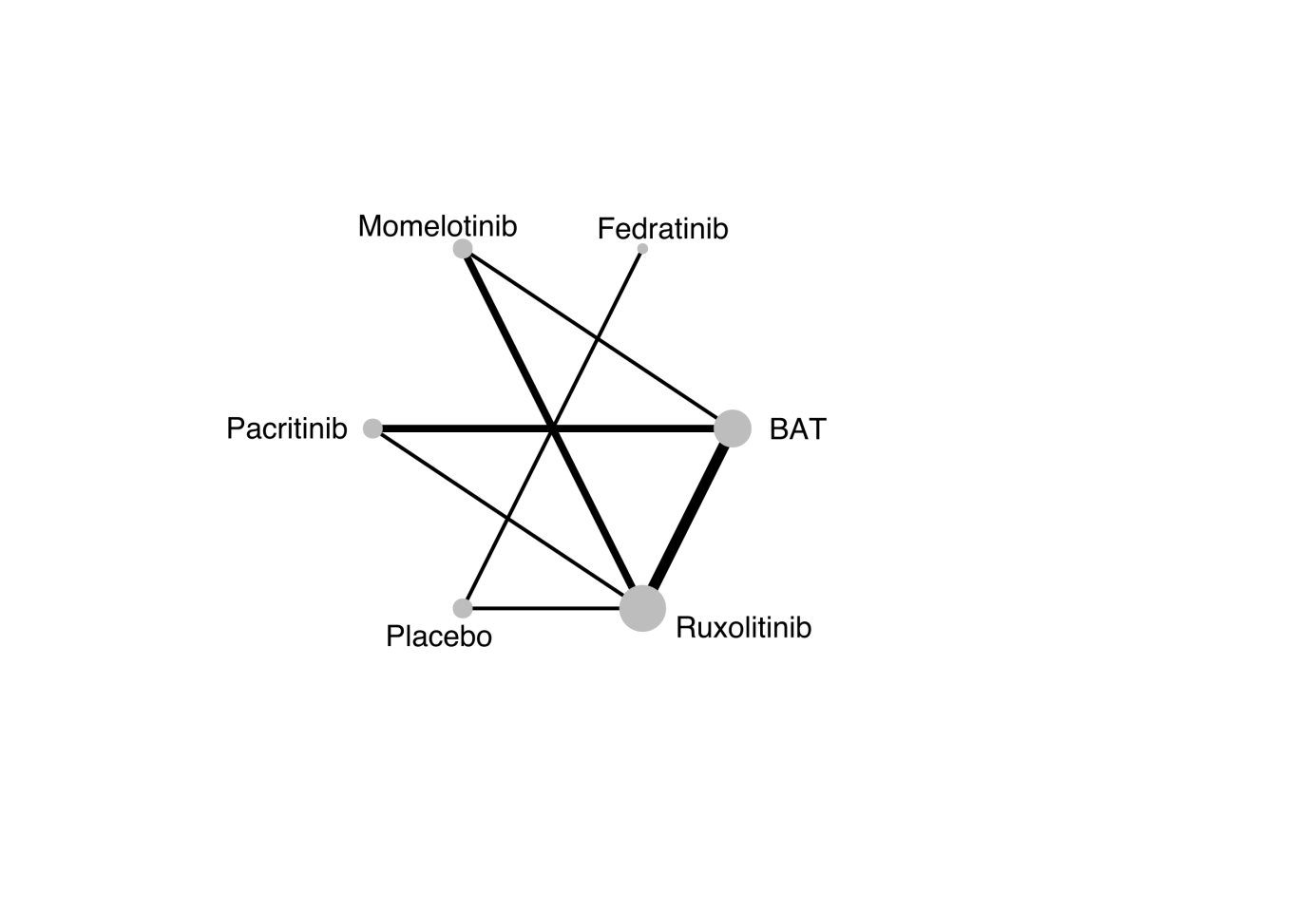


**Figure S2**: Geometry of the network for the meta-analysis

Node size is proportional to the total number of patients for each arm in the network, edge width is proportional to the number of trials of direct comparisons between treatments.

BAT: Best available treatment, in this figure is strictly different from anti-JAK 2.

# Summary of the risk of bias in the included studies

The risk of bias of the systematic review and network meta-analysis was assessed using five criteria (Figure S3). The source of bias associated with the higher risk was the “allocation concealment” due to the inclusion of four out of seven trials in open label. The overall risk of bias was nonetheless estimated as low.

**Figure S3**: Summary of the risk of bias in the studies included in the systematic review

ITT: intention-to-treat

# Preliminary and sensitivity tests for Spleen Volume Reduction (SVR)

A preliminary test was done to estimate the inconsistency within and between the seven studies SVR data. Results were presented as the I^2^ indicator and the funnel plot (Figure S4). It was decided to exclude trials using second-line anti-JAK2 from the primary analysis. However, a sensitivity analysis including all studies was done (Figure S5). It showed that pacritinib results were drastically improved when included its use in second line after ruxolitinib. Regarding momelotinib, the inclusion of second-line data did not impact the results.


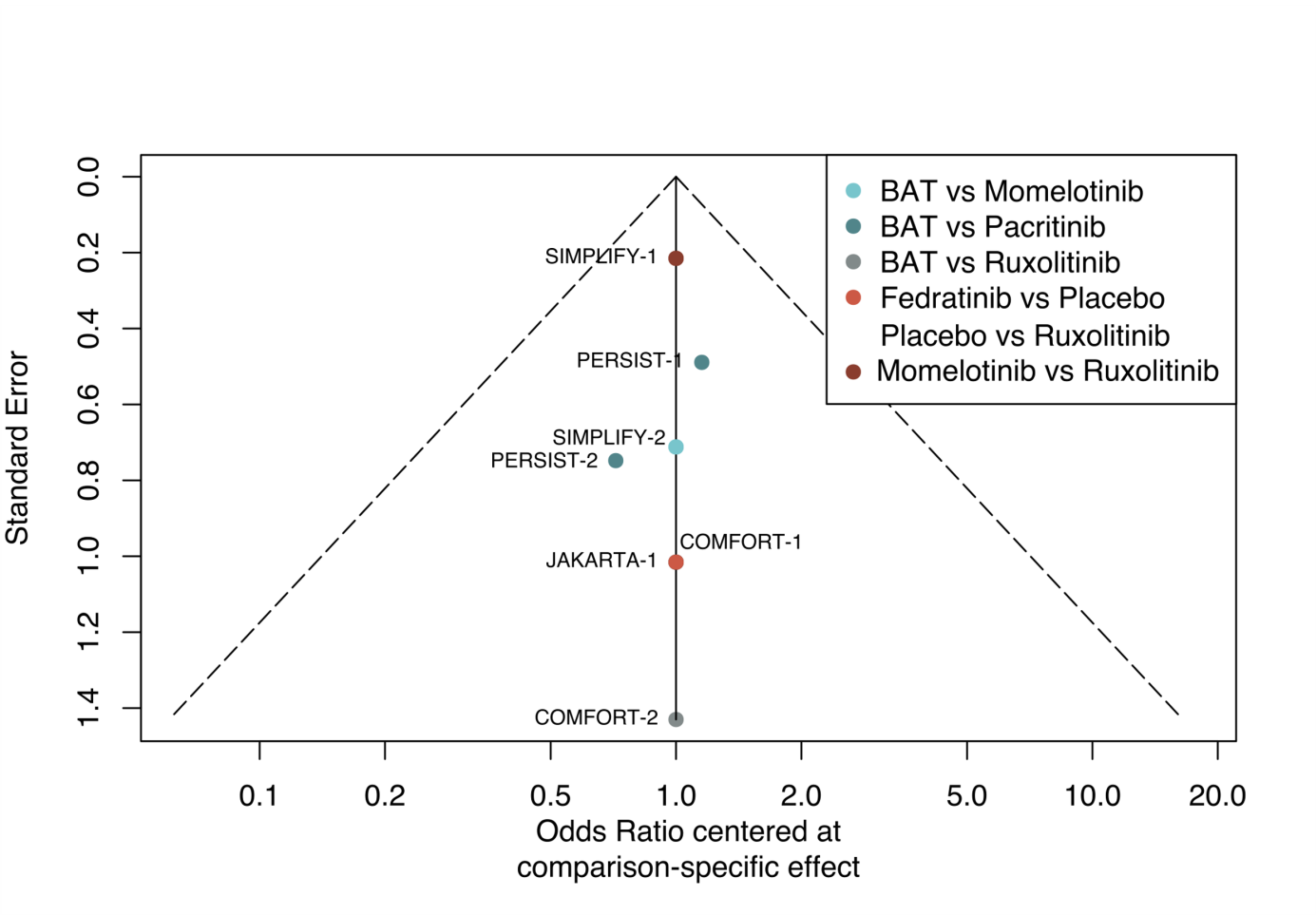


**Figure S4**: Funnel plot for SVR analysis with the seven trials included in the systematic review

BAT: Best available treatment


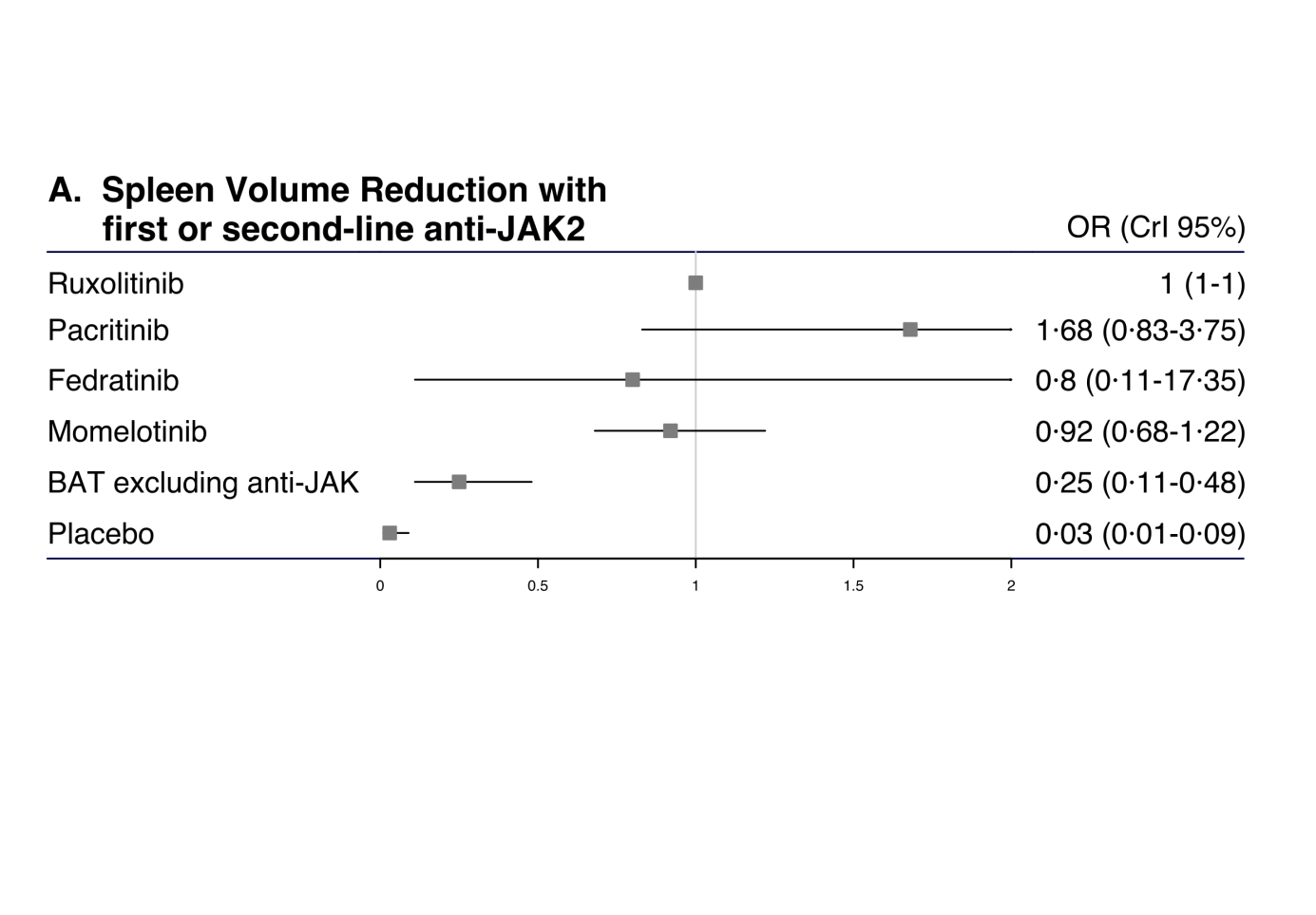


**Figure S5**: Estimates of risk in first and second lines in the intention-to-treat population for spleen volume reduction

# Sensitivity tests and additional analysis for grade 3/4 anemia events

Two of the seven trials presented baseline hemoglobin rates different from the mean rate in the global population of the analysis (Table S3). In consequence, it was decided to do two sensitivity tests, each of them excluding one of the two incriminated trials: the first one being SIMPLIFY-2 (Figure S6-A) where the mean hemoglobin rate at baseline was significatively lower, and the second-one PERSIST-1 (Figure S6-B) where the mean hemoglobin rate at baseline was significatively higher.

Two of the seven trials – i.e. PERSIST-2 and SIMPLIFY-2 – included data obtained with second-line therapies and were excluded in a sensitivity test analyzing only first-line therapies (Figure S6-C). This test confirmed the results obtained with the main analysis.

An additional network meta-analysis was performed and presented (Figure S6-D) has it shows that fedratinib at a 400 mg daily dose is associated with less cases of anemia grade 3 and 4 than fedratinib 500 mg daily. Although this test did not permit to show any significant difference between one of these two dosages of fedratinib and ruxolitinib.


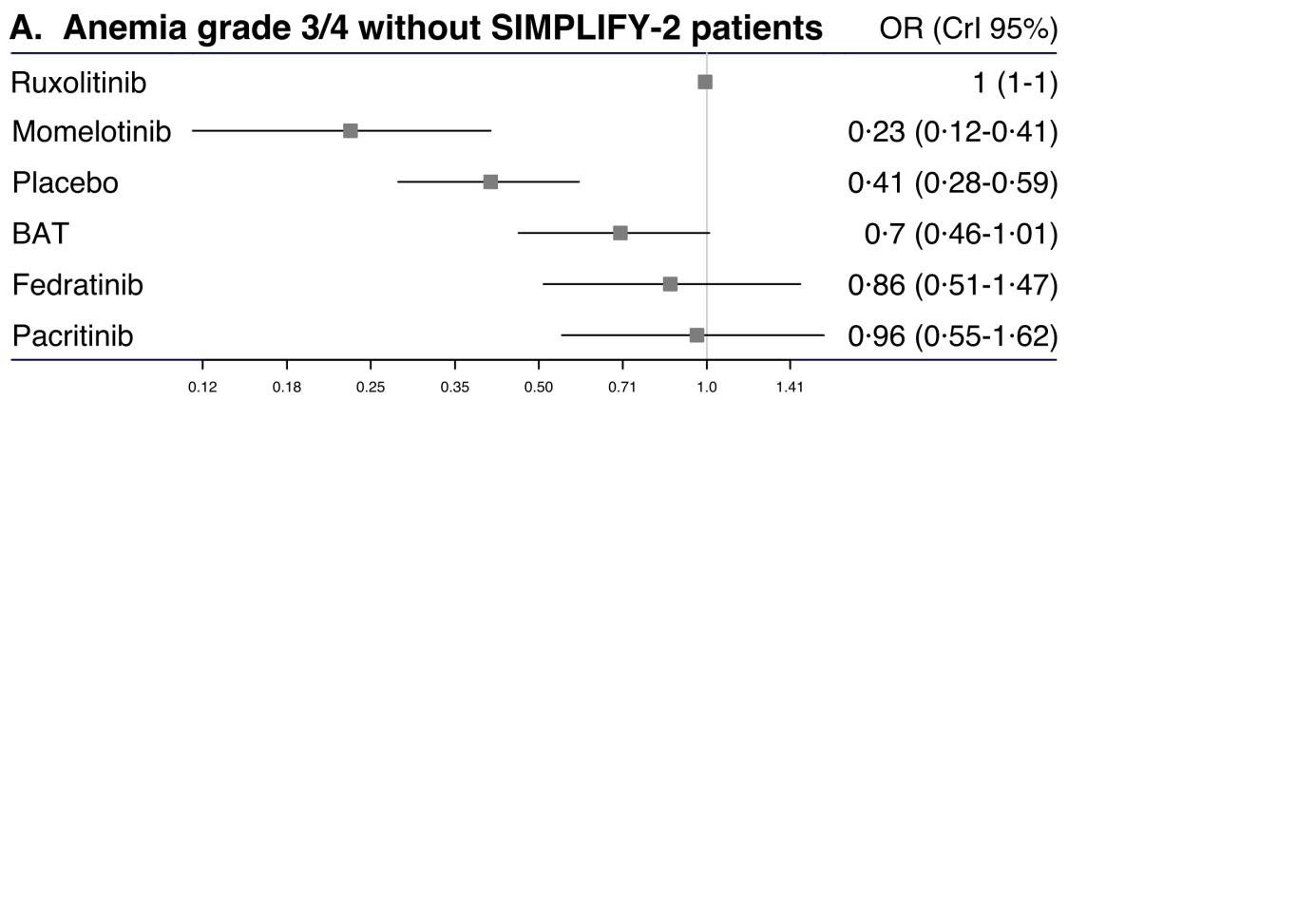

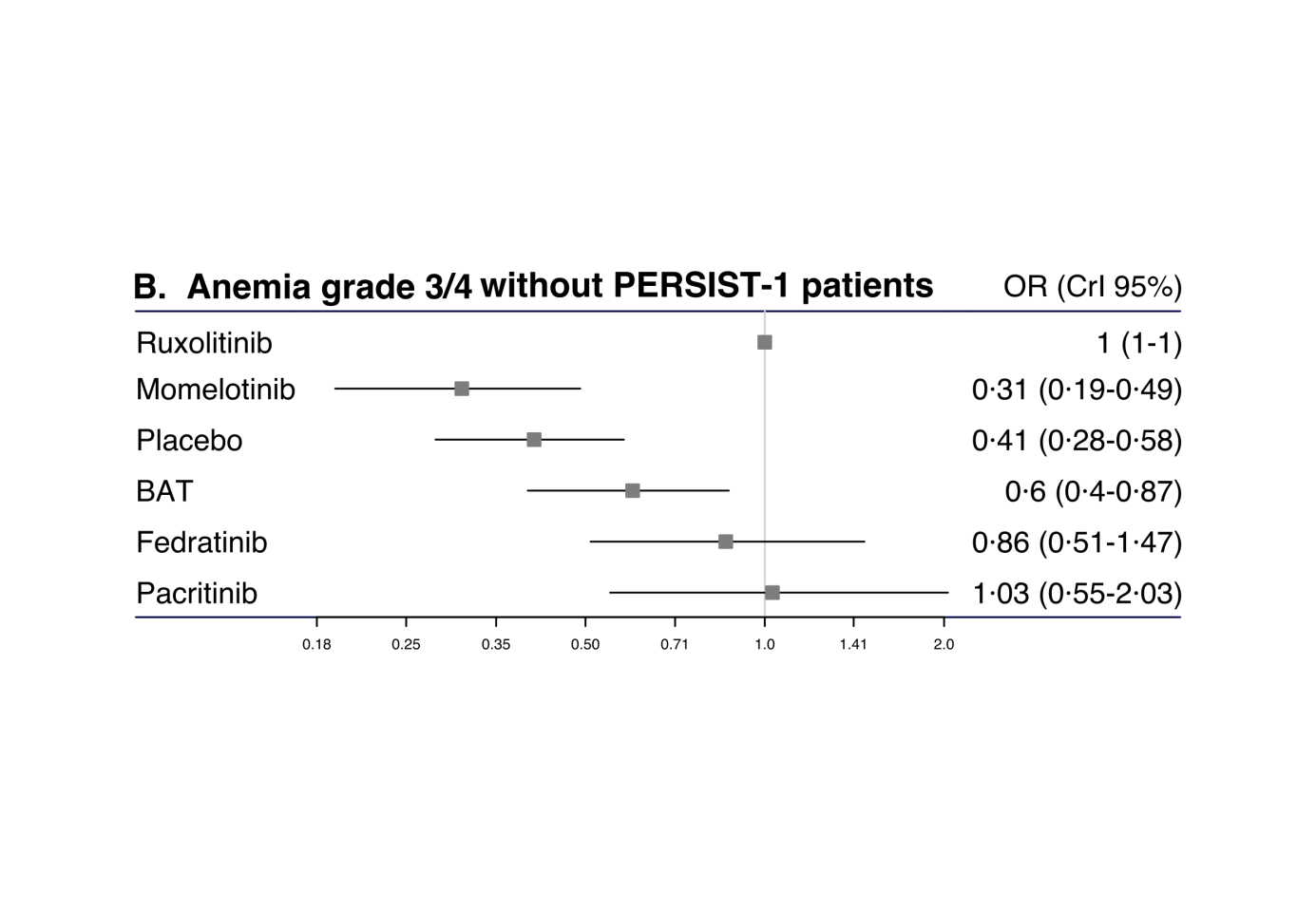


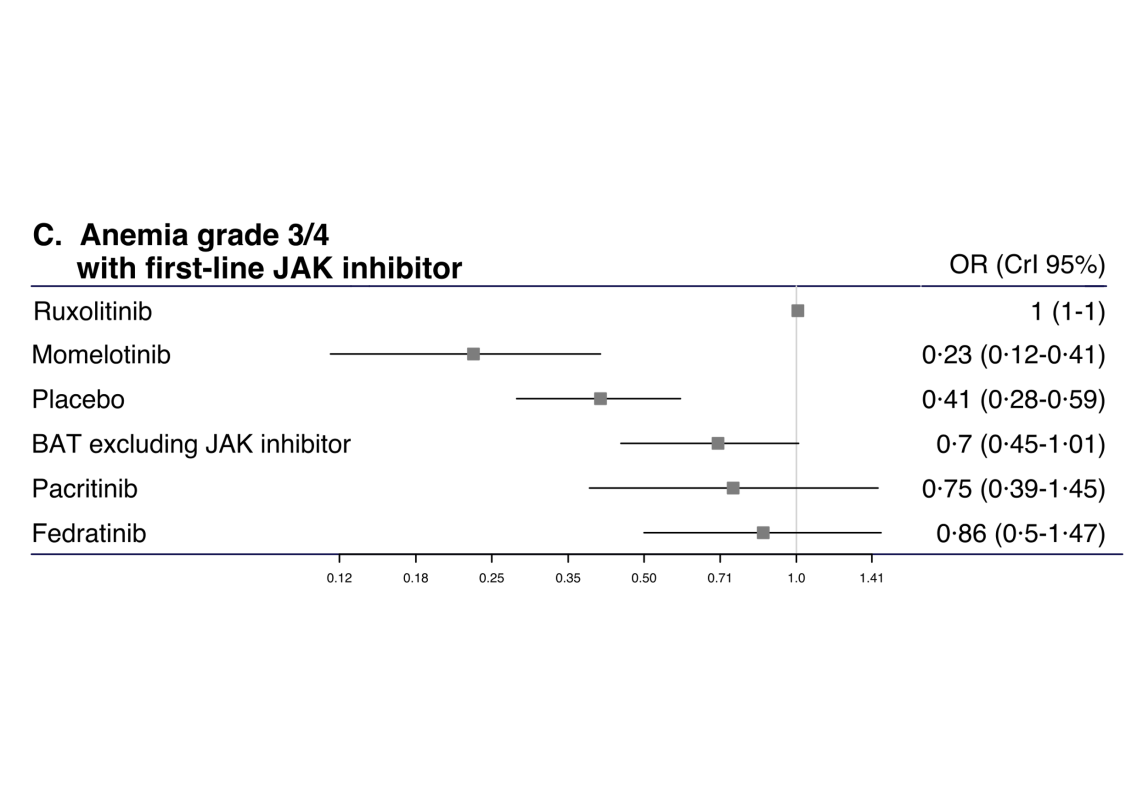


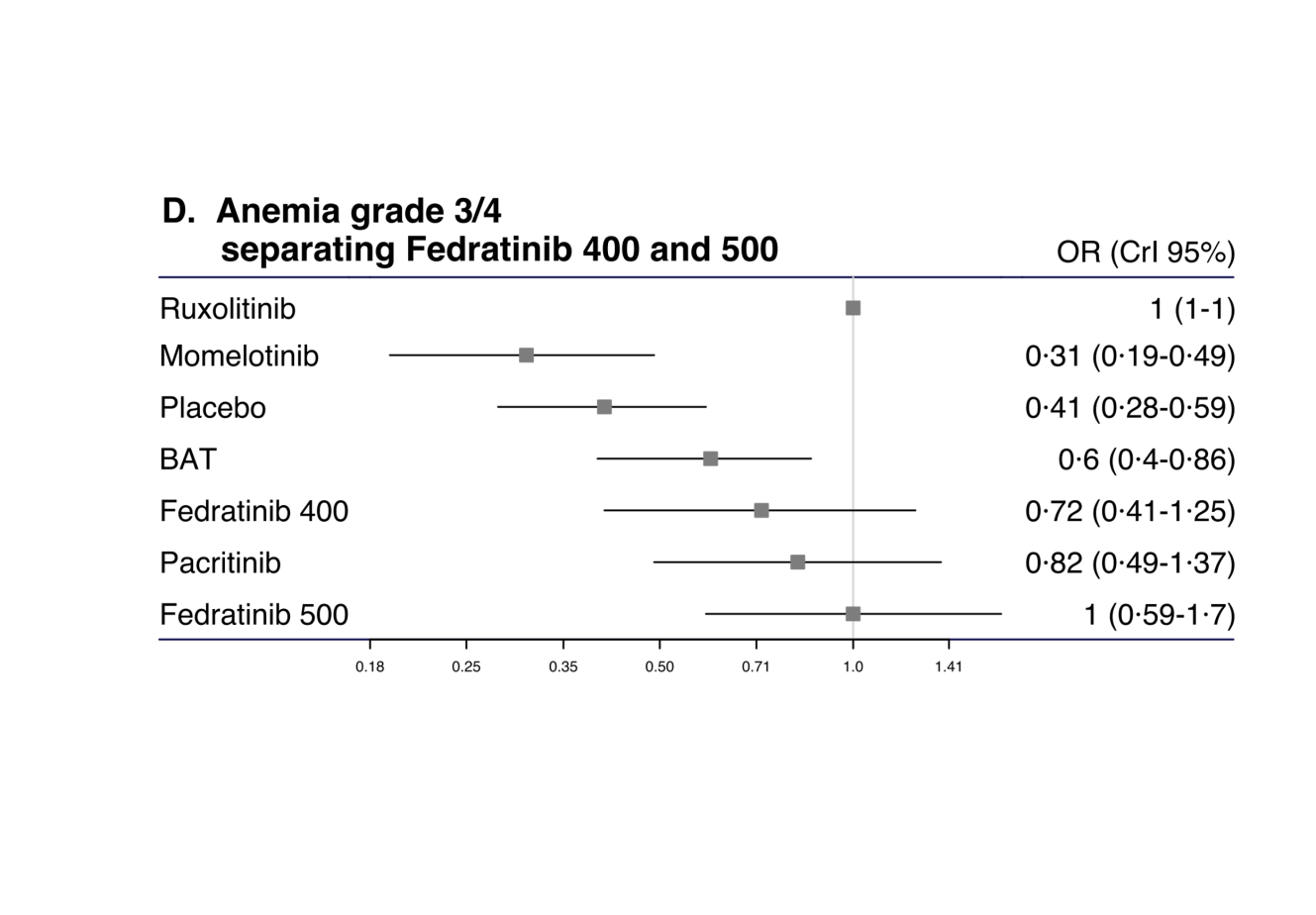


**Figure S6**: Estimates of risk in the intention-to-treat population for grade 3/4 anemia events, which (A) excludes data from SIMPLIFY-2 because of a lower overall baseline hemoglobin rate, (B) excludes data from PERSIST-1 because of a higher overall baseline hemoglobin rate, (C) excludes data from PERSIST-2 and SIMPLIFY-2 to analyze only first-line therapies, (D) distinguishes between fedratinib 400 mg and fedratinib 500 mg daily.

# Sensitivity test for grade 3/4 thrombopenia events

Three of the seven studies included in the network meta-analysis did not have thrombocytopenia – platelets < 50.10^9^/L- at baseline as an exclusion criterion (Table S4). For this reason, a sensitivity test was performed excluding the three incriminated studies, i.e. SIMPLIFY-2, PERSIST-1 and PERSIST-2, to ensure that results obtained with the primary analysis were not influenced by the baseline characteristics of patients (Figure S7). Consequently, this sensitivity test was performed using data obtained with first-line therapies only.

Momelotinib and Pacritinib were potentially affected in the sensitivity test. This one did not permit to conclude on pacritinib as it excluded all patients treated by this drug. Regarding momelotinib, the sensitivity test confirmed it was associated to more thrombopenia events than other anti-JAK2, even if the analysis did not highlight any statistically significant difference. This test also confirmed the superiority of fedratinib.


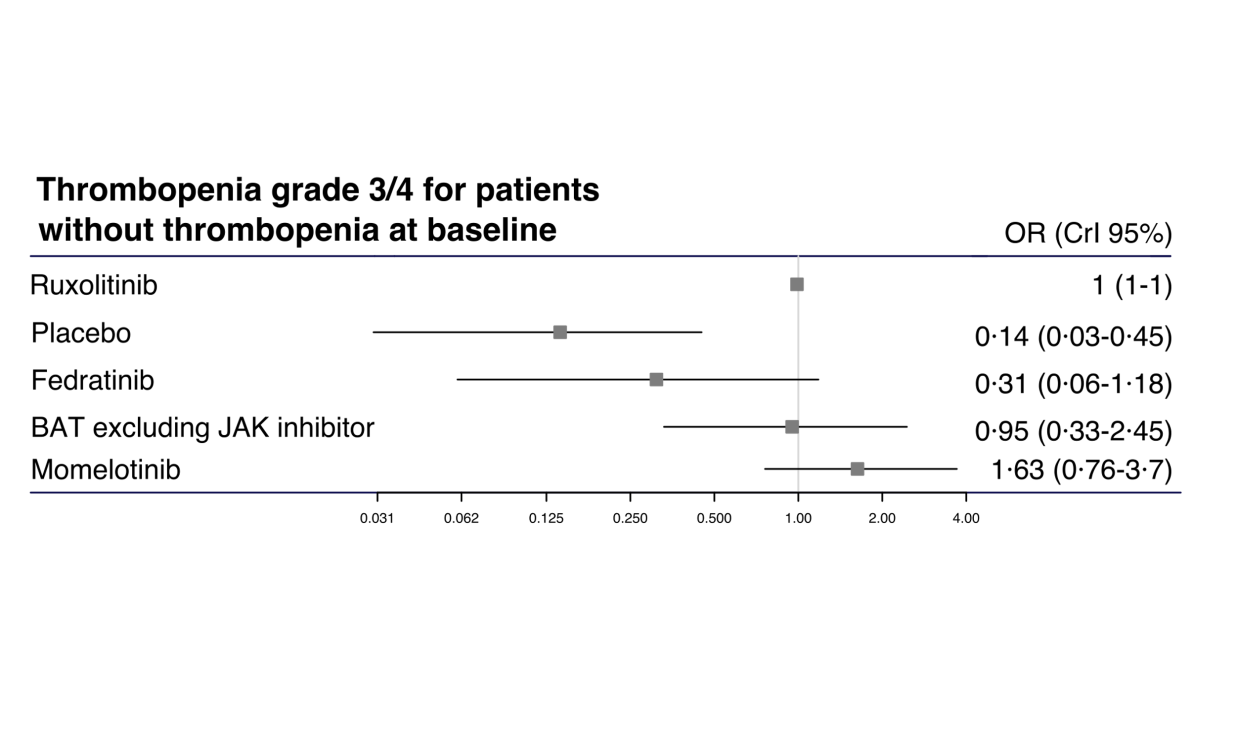


**Figure S7**: Estimates of risk with first-line JAK inhibitor in the intention-to-treat population for grade 3/4 thrombopenia events, without studies that included patients with thrombopenia at baseline

# Study screening and inclusion in the systematic review and meta-analysis

**Table S1**: Search strategy for study screening

| **Database** | **Search strategy** | **Accessed on (date)** |
| --- | --- | --- |
| In Clinicaltrials.gov database | Condition: “myelofibrosis”  Other terms: “JAK2 inhibitor OR JAK1 inhibitor OR Janus kinase OR JAK”  Status: “Active, not recruiting”; “Suspended”; “Terminated”; “Completed”; “Withdrawn”; “Unknown status " | April 12, 2021 |
| In CENTRAL database | in Trials section  "Myelofibrosis" and "Janus kinase inhibitor OR JAK" in Abstract | April 12, 2021 |
| In PubMed database | janus kinase OR jak AND spleen reduction AND myelofibrosis AND (Clinical Trial[ptyp])  Search details: ("janus kinases"[MeSH Terms] OR ("janus"[All Fields] AND "kinases"[All Fields]) OR "janus kinases"[All Fields] OR ("janus"[All Fields] AND "kinase"[All Fields]) OR "janus kinase"[All Fields]) OR jak[All Fields] AND (("spleen"[MeSH Terms] OR "spleen"[All Fields]) AND reduction[All Fields]) AND ("primary myelofibrosis"[MeSH Terms] OR ("primary"[All Fields] AND "myelofibrosis"[All Fields]) OR "primary myelofibrosis"[All Fields] OR "myelofibrosis"[All Fields]) AND Clinical Trial[ptyp] | April 12, 2021 |

**Table S2**: Registration numbers of trials of anti-JAK2 efficacy and tolerance meeting inclusion criteria

|  | NCT number | Study title |
| --- | --- | --- |
| COMFORT-1 | NCT00952289 | COntrolled MyeloFibrosis Study With ORal JAK Inhibitor Treatment: The COMFORT-I Trial |
| COMFORT-2 | NCT00934544 | Controlled Myelofibrosis Study With Oral Janus-associated Kinase (JAK) Inhibitor Treatment-II: The COMFORT-II Trial |
| JAKARTA-1 | NCT00934544 | Phase III Study of SAR302503 in Intermediate-2 and High Risk Patients With Myelofibrosis |
| PERSIST-1 | NCT01773187 | Oral Pacritinib Versus Best Available Therapy to Treat Myelofibrosis |
| PERSIST-2 | NCT02055781 | Oral Pacritinib Versus Best Available Therapy to Treat Myelofibrosis With Thrombocytopenia |
| SIMPLIFY-1 | NCT01969838 | Momelotinib Versus Ruxolitinib in Subjects With Myelofibrosis |
| SIMPLIFY-2 | NCT02101268 | Efficacy of Momelotinib Versus Best Available Therapy in Anemic or Thrombocytopenic Subjects With Primary Myelofibrosis (MF), Post-polycythemia Vera MF, or Post-essential Thrombocythemia MF |

# PRISMA checklist for systematic review or meta-analysis

**Table S3**: The PRISMA extension statement for reporting of systematic reviews incorporating network meta-analyses of health care interventions: checklist of items to include when reporting a systematic review or meta-analysis

| Section/topic | # | Checklist item | Reported on page # of the manuscript |
| --- | --- | --- | --- |
| **TITLE** | | | |
| Title | 1 | Identify the report as a systematic review, meta-analysis or both. | 1 |
| **ABSTRACT** | | | |
| Structured summary | 2 | ﻿Provide a structured summary including, as applicable: Background: main objectives Methods: data sources; study eligibility criteria, participants, and interventions; study appraisal; and synthesis methods, such as network meta-analysis.  Results: number of studies and participants identified; summary estimates with corresponding confidence/credible intervals; treatment rankings may also be discussed. Authors may choose to summarize pairwise comparisons against a chosen treatment included in their analyses for brevity.  Discussion/Conclusions: limitations; conclusions and implications of findings. Other: primary source of funding; systematic review registration number with registry name. | 2 |
| **INTRODUCTION** | | | |
| Rationale | 3 | ﻿Describe the rationale for the review in the context of what is already known, including mention of why a network meta-analysis has been conducted. | 3 |
| Objectives | 4 | ﻿Provide an explicit statement of questions being addressed, with reference to participants, interventions, comparisons, outcomes, and study design (PICOS). | 3 |
| **METHODS** | | | |
| Protocol and registration | 5 | ﻿Indicate whether a review protocol exists and if and where it can be accessed (e.g., Web address); and, if available, provide registration information, including registration number. | NA |
| Eligibility criteria | 6 | ﻿Specify study characteristics (e.g., PICOS, length of follow -up) and report characteristics (e.g., years considered, language, publication status) used as criteria for eligibility, giving rationale. Clearly describe eligible treatments included in the treatment network and note whether any have been clustered or merged into the same node (with justification). | 3-4 |
| Information sources | 7 | ﻿Describe all information sources (e.g., databases with dates of coverage, contact with study authors to identify additional studies) in the search and date last searched. | 4 and SM |
| Search | 8 | ﻿Present full electronic search strategy for at least one database, including any limits used, such that it could be repeated. | SM |
| Study selection | 9 | ﻿State the process for selecting studies (i.e., screening, eligiblity, included in systematic review, and, if applicable, included in the meta-analysis). | SM |
| Data collection process | 10 | ﻿Describe method of data extraction from reports (e.g., piloted forms, independently, in duplicate) and any processes for obtaining and confirming data from investigators | 4 |
| Data items | 11 | List and define all variables for which data were sought (e.g., PICOS, funding sources) and any assumptions and simplifications made. | x |
| Geometry of the network | S1 | ﻿Describe methods used to explore the geometry of the treatment network under study and potential biases related to it. This should include how the evidence base has been graphically summarized for presentation, and what characteristics were compiled and used to describe the evidence base to readers. | SM |
| Risk of bias in individual studies | 12 | Describe methods used for assessing risk of bias of individual studies (including specification of whether this was done at the study or outcome level), and how this information is to be used in any data synthesis. | SM |
| Summary measures | 13 | State the principal summary measures (e.g., risk ratio, difference in means). | 4 |
| Synthesis of results | 14 | Describe the methods of handling data and combining results of studies, if done, including measures of consistency (e.g., I^2^) for each meta-analysis. | 5 |
| Assessment of inconsistency | S2 | ﻿Describe the statistical methods used to evaluate the agreement of direct and indirect evidence in the treatment network(s) studied. Describe efforts taken to address its presence when found. | 5 |
| Risk of bias across studies | 15 | Specify any assessment of risk of bias that may affect the cumulative evidence (e.g., publication bias, selective reporting within studies). | SM |
| Additional analyses | 16 | Describe methods of additional analyses (e.g., sensitivity or subgroup analyses, meta-regression), if done, indicating which were pre-specified. | 5 |
| **RESULTS** | | | |
| Study selection | 17 | Give numbers of studies screened, assessed for eligibility, and included in the review, with reasons for exclusions at each stage, ideally with a flow diagram. | SM |
| Presentation of network structure | S3 | Provide a network graph of the included studies to enable visualization of the geometry of the treatment network. | SM |
| Summary of network geometry | S4 | ﻿Provide a brief overview of characteristics of the treatment network. This may include commentary on the abundance of trials and randomized patients for the different interventions and pairwise comparisons in the network, gaps of evidence in the treatment network, and potential biases reflected by the network structure. | 6 |
| Study characteristics | 18 | For each study, present characteristics for which data were extracted (e.g., study size, PICOS, follow-up period) and provide the citations. | 5, 6 |
| Risk of bias within studies | 19 | Present data on risk of bias of each study and, if available, any outcome-level assessment (see Item 12). | SM |
| Results of individual studies | 20 | For all outcomes considered (benefits or harms), present, for each study: (a) simple summary data for each intervention group and (b) effect estimates and confidence intervals, ideally with a forest plot. | 6-8 |
| Synthesis of results | 21 | Present results of each meta-analysis done, including confidence intervals and measures of consistency. | 6-8 |
| Exploration for inconsistency | S5 | ﻿Describe results from investigations of inconsistency. This may include such information as measures of model fit to compare consistency and inconsistency models, P values from statistical tests, or summary of inconsistency estimates from different parts of the treatment network. | 6-8, SM |
| Risk of bias across studies | 22 | Present results of any assessment of risk of bias across studies (see Item 15). | 6-8, SM |
| Additional analysis | 23 | Give results of additional analyses, if done (e.g., sensitivity or subgroup analyses, meta-regression [see Item 16]). | 6-8, SM |
| **DISCUSSION** | | | |
| Summary of evidence | 24 | Summarize the main findings including the strength of evidence for each main outcome; consider their relevance to key groups (e.g., health care providers, users, and policy makers). | 8-11 |
| Limitations | 25 | Discuss limitations at study and outcome level (e.g., risk of bias), and at review level (e.g., incomplete retrieval of identified research, reporting bias). | 8-11 |
| Conclusions | 26 | Provide a general interpretation of the results in the context of other evidence, and implications for future research. | 8-11 |
| **FUNDING** | | | |
| Funding | 27 | Describe sources of funding for the systematic review and other support (e.g., supply of data); role of funders for the systematic review. | NA |

# Baseline repartition depending on hemoglobin rate and platelet counts

**Table S4**: Patients repartition at baseline per hemoglobin rates

|  | |  |  |  | Distribution, n (%) | | | |  |
| --- | --- | --- | --- | --- | --- | --- | --- | --- | --- |
| Study and arm | *Population* | | *Median (range) hemoglobin level* | *Mean (SD) hemoglobin level* | **< 8 g/dL** | **≥ 8 g/dL** | **< 10 g/dL** | **≥ 10 g/dL** | |
| JAKARTA 1 fedratinib 400 mg | | 96 | 10·7 (4·8-16·8) |  |  |  |  |  | |
| JAKARTA 1 fedratinib 500 mg | | 97 | 9·8 (5·0-17·4) |  |  |  |  |  | |
| JAKARTA 1 placebo | | 96 | 10·1 (4·5-17·1) |  |  |  |  |  | |
| SIMPLIFY 1 momelotinib | | 215 |  | 10·6 (2·10) | 29 (13·49) | 186 (86·51) |  |  | |
| SIMPLIFY 1 ruxolitinib | | 217 |  | 10·7 (2·38) | 22 (10·14) | 195 (89·86) |  |  | |
| SIMPLIFY 2 momelotinib | | 104 |  | 9·4 (1·9) | 27 (25·96) | 77 (74·04) |  |  | |
| SIMPLIFY 2 BAT including ruxolitinib | | 52 |  | 9·5 (1·6) | 6 (11·54) | 46 (89·46) |  |  | |
| COMFORT I ruxolitinib | | 155 | 10·5 (6·6-17·0) |  |  |  |  |  | |
| COMFORT I placebo | | 154 | 10·5 (3·5-17·3) |  |  |  |  |  | |
| COMFORT 2 ruxolitinib | | 146 |  |  |  |  | 66 (45·21) | 80 (54·79) | |
| COMFORT 2 BAT | | 73 |  |  |  |  | 38 (52·05) | 35 (47·95) | |
| PERSIST 1 pacritinib | | 220 |  |  |  |  | 84 (38·18) | 136 (61·82) | |
| PERSIST 1 BAT | | 107 |  |  |  |  | 47 (43·93) | 59 (55·14) | |
| PERSIST 2 pacritinib 400 once daily | | 75 |  |  |  |  | 45 (60) | 30 (40) | |
| PERSIST 2 pacritinib 200 twice daily | | 74 |  |  |  |  | 44 (59·46) | 30 (40·54) | |
| PERSIST 2 BAT including ruxolitinib | | 72 |  |  |  |  | 41 (56·94) | 31 (43·06) | |

BAT: Best Available Therapy.

**Table S5**: Patients repartition at baseline per platelet counts

|  | |  |  |  | Distribution, n (%) | | |
| --- | --- | --- | --- | --- | --- | --- | --- |
| Study and arm | *Population* | | *Median (range) platelet count* | *Mean (SD) platelet count* | **< 50 x 10^9 /L** | **50 to <100 x 10^9 /L** | **≥ 100 x 10^9 /L** |
| JAKARTA 1 fedratinib 400 mg* | | 96 | 221 (31-1155) |  | 0 |  | 82 (85·42) |
| JAKARTA 1 fedratinib 500 mg* | | 97 | 241 (23-873) |  | 0 | 15 (15·46) | 82 (84·54) |
| JAKARTA 1 placebo* | | 96 | 187 (52-1075) |  | 0 | 19 (19·79) | 77 (80·21) |
| SIMPLIFY 1 momelotinib* | | 215 |  | 301·1 (207·03) | 0 |  |  |
| SIMPLIFY 1 ruxolitinib* | | 217 |  | 301·5 (255·88) | 0 |  |  |
| SIMPLIFY 2 momelotinib | | 104 |  | 170·8 (148·0) |  |  |  |
| SIMPLIFY 2 BAT including ruxolitinib | | 52 |  | 126·5 (95·9) |  |  |  |
| COMFORT I ruxolitinib^†^ | | 155 | 262 (81-984) |  | 0 | 0 | 155 (100) |
| COMFORT I placebo^†^ | | 154 | 238 (100-887) |  | 0 | 0 | 154 (100) |
| COMFORT 2 ruxolitinib | | 146 | 244 |  |  |  |  |
| COMFORT 2 BAT | | 73 | 228 |  |  |  |  |
| PERSIST 1 pacritinib | | 220 |  |  | 35 (15·91) | 37 (16·82) | 148 (67·27) |
| PERSIST 1 BAT | | 107 |  |  | 16 (14·95) | 18 (16·82) | 73 (68·22) |
| PERSIST 2 pacritinib 400 once daily^‡^ | | 75 |  |  | 38 (50·67) | 37 (49·33) | 0 |
| PERSIST 2 pacritinib 200 twice daily^‡^ | | 74 |  |  | 31 (41·89) | 43 (58·11) | 0 |
| PERSIST 2 BAT including ruxolitinib^‡^ | | 72 |  |  | 32 (44·44) | 40 (55·56) | 0 |

BAT: Best Available Therapy. *Platelet count ≥ 50.10^9^/L at baseline was an inclusion criterion – or < 50.10^9^/L an exclusion criterion; ^†^Platelet count < 100/10^9^/L was an exclusion criterion; ^‡^Platelet count > 100/10^9^/L was an exclusion criterion.

# Adverse events of JAK inhibitors

**Table S6**: Most common JAKi adverse events (AE) when used in myelofibrosis

|  | Ruxolitinib | | Fedratinib | | Momelotinib^a^ | | Pacritinib | |
| --- | --- | --- | --- | --- | --- | --- | --- | --- |
|  | n=301 | | n=96 | | n=318 | | n=430 | |
| Grades | All | 3 or 4 | All | 3 or 4 | All | 3 or 4 | All | 3 or 4 |
| **Hematologic** |  |  |  |  |  |  |  |  |
| Anemia | 96% | 44% | 99% | 43% | 15% | 8% | 25% | 21% |
| Thrombocytopenia | 69% | 11% | 63% | 17% | 17% | 7% | 25% | 21% |
| Neutropenia | 19%^b^ | 7%^b^ | 28% | 8% | *NA* | *NA* | *NA* | 8%^e^ |
| Lymphopenia | *NA* | *NA* | 57% | 21% | *NA* | *NA* | *NA* | *NA* |
| Leukopenia | *NA* | *NA* | 47% | 6% | *NA* | *NA* | 3%^f^ | 2%^f^ |
| **Nonhematologic** |  |  |  |  |  |  |  |  |
| Bruising | 19%^b^ | <1%^b^ | *NA* | *NA* | *NA* | *NA* | *NA* | *NA* |
| Bleeding | 24%^b^ | *NA* | *NA* | *NA* | *NA* | *NA* | 39%^e^ | 10%^e^ |
| Epistaxis | 6%^b^ | 0%^b^ | *NA* | *NA* | 8%^d^ | 0%^d^ | 11%^e^ | 3%^e^ |
| Infections and infestations | *NA* | *NA* | 42% | 2% | *NA* | *NA* | *NA* | *NA* |
| Urinary tract infection | 9%^b^ | 0%^b^ | 8% | *NA* | 11%^d^ | 2%^d^ | *NA* | *NA* |
| Nasopharyngitis | 16%^c^ | 0%^c^ | *NA* | *NA* | *NA* | *NA* | *NA* | *NA* |
| Pyrexia | 12% | 1% | *NA* | *NA* | 14%^d^ | 2%^d^ | 9% | 2%^f^ |
| Asthenia | 18%^c^ | 1%^c^ | 9% | 2% | 19%^d^ | 5%^d^ | *NA* | *NA* |
| Cough | 14%^c^ | 0%^c^ | *NA* | *NA* | 17%^d^ | 0%^d^ | *NA* | *NA* |
| Dyspnea | 17% | <1% | 8% | 0% | 13%^d^ | 2%^d^ | *NA* | *NA* |
| Pneumonia | *NA* | *NA* | *NA* | *NA* | *NA* | *NA* | 4%^f^ | 3% |
| Headache | 13% | <1% | *NA* | *NA* | 17% | <1%^d^ | *NA* | *NA* |
| Fatigue | 19% | 3% | 16% | 6% | 15% | <1%^d^ | 14% | 3% |
| Dizziness | 18%^b^ | <1%^b^ | *NA* | *NA* | 16% | 0%^d^ | 15%^e^ | *NA* |
| Weight decrease | *NA* | *NA* | 4% | 0% | *NA* | *NA* | *NA* | *NA* |
| Decreased appetite | *NA* | *NA* | *NA* | *NA* | *NA* | *NA* | 5%^f^ | <1%^f^ |
| Peripheral Edema | 20% | 0% | *NA* | *NA* | 10%^d^ | 0%^d^ | 12% | <1% |
| Bone pain | *NA* | *NA* | *NA* | *NA* | 2%^d^ | 0%^d^ | *NA* | *NA* |
| Pain in extremity | 12% | <1% | *NA* | *NA* | *NA* | *NA* | *NA* | *NA* |
| Back pain | 10%^c^ | 2%^c^ | *NA* | *NA* | *NA* | *NA* | *NA* | *NA* |
| Arthralgia | 12% | 1% | *NA* | *NA* | *NA* | *NA* | *NA* | *NA* |
| Insomnia | *NA* | *NA* | *NA* | *NA* | *NA* | *NA* | *NA* | *NA* |
| Pruritus | 5%^c^ | 0%^c^ | *NA* | *NA* | 13%^d^ | <1%^d^ | *NA* | *NA* |
| Hypertension | *NA* | *NA* | *NA* | *NA* | *NA* | *NA* | 3%^f^ | 2%^f^ |
| QT prolonged | *NA* | *NA* | *NA* | *NA* | *NA* | *NA* | 5% | <1% |
| Cardiac failure | *NA* | *NA* | *NA* | *NA* | *NA* | *NA* | 3% | 2% |
| Atrial fibrillation | *NA* | *NA* | *NA* | *NA* | *NA* | *NA* | 2% | 1% |
| Ejection fraction decreased | *NA* | *NA* | *NA* | *NA* | *NA* | *NA* | <1%^e^ | <1%^e^ |
| Palpitations | *NA* | *NA* | *NA* | *NA* | *NA* | *NA* | 1%^e^ | 0%^e^ |
| **Digestive** |  |  |  |  |  |  |  |  |
| Diarrhea | 23% | 2% | 66% | 5% | 23% | 3% | 56% | 5% |
| Nausea | 14% | <1% | 64% | 0% | 17% | 2%^d^ | 31% | <1%^f^ |
| Vomiting | 13%^b^ | <1%^b^ | 42% | 3% | *NA* | *NA* | 18% | <1%^f^ |
| Abdominal Pain | 10% | 3% | 15% | 0% | 12% | <1%^d^ | 12% | 1%^c^ |
| Constipation | 13%^b^ | 0%^b^ | 10% | 2% | 12%^d^ | 0%^d^ | *NA* | *NA* |

^a^Momelotinib and Pacritinib described event are treatment-emergent adverse events TEAE), meaning that these AEs represented a worsening compared to baseline and appeared after treatment initiation. ^b,c,d,e,f^Adverse events reported on 155, 146, 104, 210 and 220 patients, respectively.

*NA*: data not available
